# Promoting ranking diversity for genomics search with relevance-novelty combined model

**DOI:** 10.1186/1471-2105-12-S5-S8

**Published:** 2011-07-27

**Authors:** Xiaoshi Yin, Zhoujun Li, Jimmy Xiangji Huang, Xiaohua Hu

**Affiliations:** 1 State Key Laboratory of Software Development Environment, Beihang University, Beijing 100191, China; 2 School of Computer Science and Engineering, Beihang University, Beijing, China; 3 Beijing Key Laboratory of Network Technology, Beihang University, Beijing, China; 4 School of Information Technology, York University, Toronto, Canada; 5 College of Information Science and Technology, Drexel University, Philadelphia, PA, USA

## Abstract

**Background:**

In the biomedical domain, the desired information of a question (query) asked by biologists usually is a list of a certain type of entities covering different aspects that are related to the question, such as genes, proteins, diseases, mutations, etc. Hence it is important for a biomedical information retrieval system to be able to provide comprehensive and diverse answers to fulfill biologists’ information needs. However, traditional retrieval models assume that the relevance of a document is independent of the relevance of other documents. This assumption may result in high redundancy and low diversity in the retrieval ranked lists.

**Results:**

In this paper, we propose a relevance-novelty combined model, named RelNov model, based on the framework of an undirected graphical model. It consists of two component models, namely the aspect-term relevance model and the aspect-term novelty model. They model the relevance of a document and the novelty of a document respectively. We show that our approach can achieve 16*.*4% improvement over the highest aspect level MAP reported in the TREC 2007 Genomics track, and 9*.*8% improvement over the highest passage level MAP reported in the TREC 2007 Genomics track.

**Conclusions:**

The proposed combination model which models aspects, terms, topic relevance and document novelty as potential functions is demonstrated to be effective in promoting ranking diversity as well as in improving relevance of ranked lists for genomics search. We also show that the use of aspect plays an important role in the model. Moreover, the proposed model can integrate various different relevance and novelty measures easily.

## Background

Current genomic research is characterized by immense volume of data, accompanied by a tremendous increase in the number of genomic and biomedical related publications. This wealth of information has led to an increasing amount of interest and need for applying information retrieval (IR) techniques to access the scientific literature in genomics and related biomedical disciplines.

Given a query, an IR system returns a ranked list of retrieved documents to users. Retrieved documents are ranked in the order of their probabilities of relevance to the query. Traditional retrieval models assume that the relevance of a document is independent of the relevance of other documents. However, in reality, this assumption may not hold. The usefulness of retrieving a document usually depends on previous ranked documents, since a user may want to see the top ranked documents concerning different aspects of his/her information need instead of reading relevant documents that only deliver redundant information. A better information retrieval system thus should return ranked lists that respect both the query-relevance and the breadth of available information.

In the biomedical domain, the desired information of a question (query) asked by biologists usually is a list of a certain type of entities covering different aspects that are related to the question [1], such as genes, proteins, diseases, mutations, etc. Hence it is important for a biomedical IR system to be able to provide comprehensive and diverse answers to fulfill biologists’ information need.

To address this problem, in the most recent TREC Genomics tracks, the “aspect retrieval” was investigated. Its purpose is to study how a biomedical retrieval system can support a user gathering information about the different aspects of a topic. We consider the aspects of a document as concepts, entities or topics contained in the document. In the Genomics tracks, biomedical IR systems were required to return relevant information at the passage level, while relevance judges not only rated the passages, but also grouped them by aspect. Aspects of a retrieved passage could be a list of named entities or MeSH terms, representing answers that cover different portions of a full answer to the query. Aspect Mean Average Precision (Aspect MAP) was defined in the Genomics tracks to capture similarities and differences among retrieved passages. It indicates how comprehensive the questions are answered. Relevant passages that do not contribute any new aspects to the aspects retrieved by higher ranked passages will not be used to accumulate Aspect MAP [2]. Therefore, Aspect MAP is a measurement for redundancy and diversity of the IR ranked list.

Our work is inspired by several recent papers that concerned with promoting diversity and novelty in the IR ranked list. Carbonell *et al.* introduced the maximal marginal relevance (MMR) method, which attempted to maximize relevance while minimizing similarity to higher ranked documents [3]. In order to measure the redundancy between documents, Zhang *et al.* presented four redundancy measures, which were “set difference”, “geometric distance”, “distributional similarity” and “a mixture model” [4]. They modeled relevance and redundancy separately. Since they focused on redundant document filtering, experiments in their study were conducted on a set of relevant documents. However, in reality, non-relevant documents are always returned by IR systems along with relevant documents. Redundancy and relevance should both be considered. Zhai *et al.* validated a subtopic retrieval method based on a risk minimization framework [5]. Their subtopic retrieval method combined the mixture model novelty measure with the query likelihood relevance ranking. More recently, a new diversity task of Web retrieval was defined in the TREC 2009 Web track [6]. Two evaluation measures, *α*-nDCG [7] and an intent-aware version of precision (IA-P) [8], both of which reward novelty and diversity, were validated in the diversity task of the 2009 Web track. Top diversity task results showed that re-ranking methods based on anchor text, sites of search results, link filtering, clustering and sub-queries suggestion were effective in Web retrieval result diversification [9-12]. However, these studies mainly focused on Web search and did not take characteristics of genomics search into account. How to promote ranking diversity in the biomedical information retrieval still need to be further investigated.

In biomedical Information retrieval, the Genomics aspect retrieval was firstly proposed in the TREC 2006 Genomics track and further investigated in the 2007 Genomics track. Many research groups joined these annual campaigns to evaluate their systems and methodologies. However, to the best of our knowledge, there is not too much previous work conducted on the Genomics aspect retrieval for promoting diversity in the ranked list. University of Wisconsin re-ranked the retrieved passages using a clustering-based approach named GRASSHOPPER to promote ranking diversity [13]. GRASSHOPPER was an alternative to MMR and variants with a principled mathematical model and strong empirical performance on artificial data set [14]. Unfortunately, for the Genomics aspect retrieval, this re-ranking method hurt their system’s performance and decreased the Aspect MAP of the original results [13]. Later in the TREC 2007 Genomics track, most teams tried to obtain the aspect level performance through their passage level results, instead of working on the aspect level retrieval directly [1,15,16]. Another study concerning with the Genomics aspect retrieval was conducted in [17]. Their experimental results demonstrated that the hidden property based re-ranking method can achieve promising performance improvements.

In our preliminary study, we showed that Wikipedia can be used as an external knowledge resource to facilitate biomedical IR [18]. However, how to combine the novelty and the relevance of a document for maximizing effectiveness of IR systems remains a challenging research question.

## Methods

### Datasets and evaluation measures

In order to evaluate the proposed approach for promoting ranking diversity in biomedical information retrieval, we use the TREC 2006 and 2007 Genomics track collection as the test corpus. It is a full-text biomedical corpus consisting of 162,259 documents from 49 genomics-related journals indexed by MEDLINE [1,2]. 28 official topics from the 2006 Genomics track and 36 official topics from the 2007 Genomics track are used as queries. Topics are in the form of questions asking for lists of specific entities that cover different portions of full answers to the topics [1,2].

There were three levels of retrieval performance that were measured in the TREC 2006 and 2007 Genomics tracks: passage retrieval, aspect retrieval and document retrieval. Each was measured by some variants of mean average precision (MAP). Passage MAP, Passage2 MAP(Passage2 MAP was defined in the TREC 2007 Genomics track, which is an alternative measure to the Passage MAP defined in the TREC 2006 Genomics track.), Aspect MAP and Document MAP were four evaluation measures corresponding to the three levels of retrieval performance. The definitions of these MAPs can be found in [2] and [1]. In this paper, we mainly focus on aspect level and passage level retrieval performance, since our objective is to promote diversity in the ranked list of retrieved passages. Moreover, aspect retrieval and passage retrieval were also the major tasks of these two Genomics tracks.

Genomics collections only present a fraction of millions of biomedical literatures indexed by MEDLINE. However, to the best of our knowledge, they are the largest and the only biomedical text collections with both manual relevance assessments and diversity evaluation available for biomedical text retrieval research so far.

### Baseline runs

For the 2007’s topics, three IR baseline runs are used. NLMinter [15] and MuMshFd [19] were two of the most competitive IR runs submitted to the TREC 2007 Genomics track. NLMinter developed by the U.S. National Library of Medicine [15] achieved the best performance in the TREC 2007 Genomics track in terms of Aspect MAP, Passage2 MAP and Document MAP [1,15]. It merged the retrieval results obtained by Essie [20], Indri, Terrier [21], Theme, and EasyIR [22] and employed a human-involved relevance feedback method. Another IR baseline run is an Okapi run, which is solely based on the probabilistic weighting model BM25 [23]. The performance of the Okapi run is also above average among all results reported in the TREC 2007 Genomics track [1].

For 2006’s topics, we test our approach on three Okapi runs since other retrieval results submitted to the TREC 2006 Genomics track are not available. In order to find out wether the proposed methods can work well on strong baselines as well as on average and weak baselines, we set different values to BM25 parameters to obtain different baselines [24]. The performance of the baseline run Okapi06b is also among the top performances reported in the TREC 2006 Genomics track [2].

The performance of baseline runs are shown in Table 1. The best and mean results reported in the 2006 and 2007 Genomics tracks are shown in Table 2.

**Table 1:** Performance of IR baseline runs

MAP	Aspect	Passage	Passage2	Document
NLMinter	0.2631	0.0968	0.1148	0.3286
MuMshFd	0.2068	0.0840	0.0895	0.2906
Okapi07	0.1428	0.0633	0.0641	0.2025

Okapi06a	0.2176	0.0362	0.0450	0.3476
Okapi06b	0.3147	0.1559	0.0968	0.4705
Okapi06c	0.2596	0.0759	0.0601	0.4388

The performance of IR baseline runs in terms of Aspect MAP, Passage MAP, Passage2 MAP and Document MAP.

**Table 2:** The best and mean results in the Genomics tracks

MAP	Best MAP		Mean MAP	
	
	2006	2007	2006	2007
Aspect	0.4411	0.2631	0.1643	0.1326
Passage	0.1486	0.0976	0.0392	0.0560
Passage2		0.1148		0.0398
Document	0.5439	0.3286	0.2887	0.1862

The best and mean results reported in TREC 2006 and 2007 Genomics track are shown in this table.

### Aspect detection

As described above, we focus on the aspect retrieval for promoting ranking diversity. Therefore, it is necessary to detect aspects contained in a document. Because of the frequent use of acronyms, the presence of homonyms and synonyms in biomedical literatures, using terms that appear in documents as aspects for re-ranking would not be effective. For example, when the term “AIDS” appears in a document, it may indicate “human immunodeficiency virus (HIV)” or “the medical helps given to patients”. Apparently, using the bag-of-word method could not capture the semantic meaning of terms. In the following, we use Wikipedia for aspect detection.

Wikipedia is a free online encyclopedia edited collaboratively by large numbers of volunteers. The exponential growth and the reliability of Wikipedia make it a potentially valuable knowledge resource. How to utilize Wikipedia to facilitate information retrieval became a hot research topic over the last few years [25-27].

However, as far as we are aware, there is no work done on investigating how to use Wikipedia to improve biomedical IR performance. The main reason for this is that some domain-specific thesauri are available for biomedical retrieval (e.g. UMLS, MeSH and the Gene Ontology). Nonetheless, these domain-specific thesauri only provide synonyms, hypernyms, hyponyms of a specific term without any other context. Therefore, it is hard to tell which lexical variants of a specific term should be used for retrieving users’ information need. Previous studies showed that lexical variants from domain-specific thesauri were usually assigned manually to achieve performance improvements [28,29]. The retrieval results of using domain-specific thesauri are somewhat conflicting [15,19,30].

Wikipedia, on the other hand, not only provides concepts (entities) and lexical variants of a specific term, but also provides abundant contexts. With the help of enriched entity pages, it is possible to identify which concepts and lexical variants are related under a specific context. As Wikipedia articles are constantly being updated and new entries are created everyday [27], we can expect that Wikipedia covers the great majority of medical terms. Another reason of using Wikipedia is that it contains plenty of linkage information among semantic related entities. Each link in Wikipedia is associated with an anchor text, which can be regarded as a descriptor of its target article. Anchor texts provide alternative names, morphological variations and related phrases for the target articles. Anchors also encode polysemy, because the same anchor may link to different articles depending on the context in which it is found [31]. Using Wikipedia for aspect detection, there are three steps involved:

(1) identifying candidate phrases in the given retrieved document;

(2) mapping them to Wikipedia articles;

(3) selecting the most salient concepts.

The outcome is a set of concepts representing the aspects mentioned in the input documents [26,31]. The Wikify service provided by the Wikipedia Miner(http://wikipedia-miner.sourceforge.net) is used to automatically detect aspects covered by retrieved documents.

An example is shown in Table 3. Terms that can be linked to their corresponding Wikipedia concepts are displayed in bold font. Although “SLE” has more than twenty distinct entities in Wikipedia, in this example, “SLE” is successfully linked to Wikipedia concept “Systemic lupus erythematosus”. This is because the contexts provided by the enriched Wikipedia entity pages help the disambiguation. Terms “lupus”, “nephritis”, “antibodies”, “serum” and “renal disease” are contained in both the “Systemic lupus erythematosus” Wikipedia page and the retrieved passage. While for other “SLE” related entities in Wikipedia, e.g. “Sober living environment” and “Supported leading edge”, we can barely find any common terms between the entity page and the retrieved passage.

**Table 3:** An example of aspect detection using Wikipedia

*query*: What serum [PROTEINS] change expression in association with high disease activity in lupus?
*retrieved passage*: The association aCL anti- GPI **lupus nephritis** strengthened association seen aCL positivity conjunction positivity **anti-dsDNA** anti-C1q **antibodies** examined presence levels **serum** act useful markers severity **renal disease** lupus nephritis patients likely positive **autoantibodies** non-nephritis **SLE** PAPS patients Further studies monitoring **autoantibodies** determining disease activity predicting development nephritis **SLE**

*detected aspects*: Lupus nephritis; Systemic lupus erythematosus; Antibody; Kidney; Autoantibody;

An example is shown in this table. Terms that can be linked to their corresponding Wikipedia concepts are displayed in bold font.

### The relevance-novelty combined model

The proposed RelNov model is based on an undirected probabilistic graphical model (Markov Random Field). A graphical model is a graph that models the joint probability distribution over a set of random variables. Nodes in the graph are a set of random variables and missing edges between nodes represent conditional independencies. The joint density can be factorized over the cliques of the graph.

**Figure 1:**
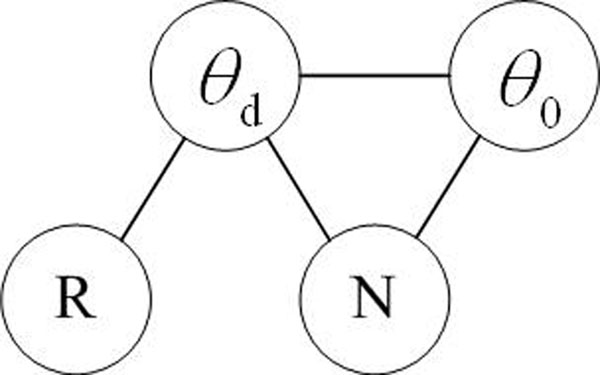
**The RelNov model**. RelNov model represents the joint probability of *θ_d_*, *θ_0_*, *R* an*d N* and edges define conditional independence assumptions between the variables.

In order to promote ranking diversity in the ranked list, we consider that the document ranking should depend on which documents the user has already seen. As shown in Figure 1, the proposed RelNov model represents the joint probability of *θ_d_*, *θ_0_*, *R* and *N*, which denote the document model of the retrieved document, the document model of previous ranked documents, the relevance of the document and the novelty of the document respectively. Edges in the graph define conditional independence assumptions between the variables. The joint distribution across potential functions in the graph represents the probability of a document being relevant to a biologist’s information need as well as being novel given the previous ranked documents.

**Figure 2:**
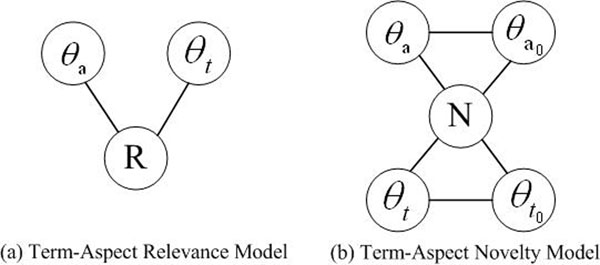
**The term-aspect relevance model and the term-aspect novelty model**. They are the two component models of the RelNov Model.

We then represent the document model *θ_d_* as *θ_t_* and *θ_a_* to capture both the lexical information and conceptual information in a retrieved document, where *θ_t_* indicates the term-based document model and *θ_a_* indicates the aspect-based document model (similarly, *θ*_*d*_0__ is presented as *θ*_*t*_0__ and *θ*_*a*_0__)*.* Since we consider the aspects of a document as concepts, entities or topics contained in the document, *θ_a_* models the conceptual information of a document. Therefore, the RelNov model can be represented as two component models shown in Figure 2, namely the term-aspect relevance model and the term-aspect novelty model.

Based on conditional independence assumptions, the joint probability distribution is written as a product of potential functions over the maximal cliques in the graph.(1)

where *ϕ*(*c*) is a positive potential function for a clique in the graph, *C* is a set of cliques in the graph, and *Z* is a normalization constant.

Potential functions in the RelNov model are defined for the cliques:(2)

As noted above, all potential functions must be non-negative, and are most commonly parameterized as:(3)

where *f*(*c*) is a real-valued feature function over clique values and *ω_c_* is the weight given to that particular feature function. Therefore, Equation (1) can be written as:(4)

In the following, we present the RelNov model’s component models, the aspect-term relevance model and the aspect-term novelty model.

### Aspect-Term relevance model

The aspect-term relevance model corresponds to the cliques *ϕ*(*θ_t_*, *R*) and *ϕ*(*θ_a_*, *R*)*.* The feature function for each clique can be written as:(5)

where *θ_u_* denotes the aspect-based document model or the term-based document model and *u_i_* denotes an aspect or a term in the document. Since we do not usually have relevance information, *P*(*u*|*R*) is unavailable. One possible solution, as introduced in [32], is to consider that *P*(*R*|*u_i_*) ≈ *P*(*Q*|*u_i_*)*.* Equation (5) thus can be re-written as:(6)

where *P*(*d_j_*|*Q*) is the relevance model presenting whether a retrieved document *d_j_* ( *j* = 1, 2, …, *N*; where *N* is the number of retrieved documents) is relevant to the query *Q*; *P*(*u_i_*|*d_j_*, *Q*) is the co-occurrence model presenting whether an aspect or a term *u_i_* is associated with the query.

The relevance model can be estimated using the baseline ranking scores of retrieved documents. To estimate the co-occurrence model, a linear interpolation of *P*(*u_i_*|*d_j_*, *Q*) and the query background information is used as:(7)

where *U* denotes the set of aspects or terms for the query; *D* denotes the set of retrieved documents for the query; *freq*(*x*, *y*) denotes the frequency of *x* in *y*; *df*(*u_i_*) denotes the document frequency of an aspect or a term *u_i_*. We use a Dirichlet prior for the smoothing parameter *µ.*(8)

where *κ* is the average aspect frequency of all aspects or terms in the retrieved documents.

### Aspect-Term novelty model

The aspect-term novelty model shown in Figure 2(b) aims to provide users with novel information instead of redundant information by promoting the diversity in the IR ranked list.

We consider that the novelty of the *i*th document depends on the *i –* 1 documents the user has already seen. Let *θ*_*u*_0__ = {*θ*_*u*_1__, *θ*_*u*_2__, …, *θ*_*u*_*i*–1__} be the aspect-based or term-based document models for previous ranked documents and *θ_u_* be the aspect-based or term-based document model for the *i*th document *d_i_*. Now, we need to measure how much novel information is contained in *d_i_*. The feature function of the aspect-term novelty model can be written as:(9)

Three obvious possibilities for combining the individual novelty scores are taking the minimum, maximum, and average. Taking the average has been shown to be more effective than taking the minimum and maximum [5]. Therefore, Equation (9) can be re-written as:(10)

Various novelty measures can be used to calculate *value_N_*(*θ_u_*;*θ*_*u*_o__)*.* In this paper, we choose the mixture model [4] as the novelty measure. Mixture model is a plausible novelty measure, which outperforms several commonly used novelty measures, e.g. set difference, KL divergency and geometry distance. It assumes that the new document is generated by a two-component mixture model, in which one component is the previous ranked document model and the other is a background language model [4][5].

## Results and Discussion

### Re-Ranking performances

In order to set the parameter values for *ω_c_*, we train *ω_c_* for 2007’s topics on 2006’s topics and train *ω_c_* for 2006’s topics on 2007’s topics by directly maximizing Aspect Mean Average Precision [33]. Note that, for each year’s topics, ∑*_c_*_∈_*_C_ω_c_* = 1 and a simple coordinate-level hill climbing search is used to optimize Aspect MAP [34]. Evaluation results of using the proposed RelNov model for document re-ranking on 2007’s topics are shown in Table 4. For 2007’s topics, *ω_c_* for each potential function in Equation (2) are set to 0.35, 0.4, 0.2 and 0.05 respectively based on the training process on 2006’s topics. The values in the parentheses are the relative rates of improvement over the original results. For 2006’s topics, re-ranking results and improvements are shown in Table 5. Based on the training process on 2007’s topics, *ω_c_* for each potential functions are set to 0.3, 0.35, 0.3 and 0.05 respectively. As we can see from these two tables, our approach achieves promising and consistent performance improvements over all baseline runs. Performance improvements can be observed on all levels of evaluation measures. It is worth mentioning that our approach can further improve the best result (NLMinter) reported in the TREC 2007 Genomics track by achieving 16*.*4% improvement on Aspect MAP and 9*.*8% improvement on Passage2 MAP. Experimental results demonstrates that our approach not only promotes diversity of ranked lists, but also improves relevance of retrieval results.

**Table 4:** Re-ranking performance on 2007’s topics with aspect detection using Wikipedia

MAP	Aspect	Passage	Passage2	Document
NLMinter	0.2631	0.0968	0.1148	0.3286
RelNov(Wiki)	0.3021	0.1020	0.1261	0.3403
	(+16.4%)	(+5.4%)	(+9.8%)	(+3.6%)

MuMshFd	0.2068	0.0840	0.0895	0.2906
RelNov(Wiki)	0.2385	0.0884	0.0939	0.3022
	(+15.3%)	(+5.2%)	(+4.9%)	(+4.0%)

Okapi07	0.1428	0.0633	0.0641	0.2025
RelNov(Wiki)	0.1679	0.0665	0.0682	0.2134
	(+17.6%)	(+5.1%)	(+6.4%)	(+5.4%)

For TREC 2007 Genomics track topics, re-ranking performances on three baseline runs in terms of Aspect MAP, Passage MAP, Passage2 MAP and Document MAP are shown in this table.

**Table 5:** Re-ranking performance on 2006’s topics with aspect detection using Wikipedia

MAP	Aspect	Passage	Passage2	Document
Okapi06a	0.2176	0.0362	0.0450	0.3476
RelNov(Wiki)	0.2341	0.0385	0.0478	0.3653
	(+7.6%)	(+6.4%)	(+6.2%)	(+5.1%)

Okapi06b	0.3147	0.1559	0.0968	0.4705
RelNov(Wiki)	0.3300	0.1619	0.1022	0.4953
	(+4.9%)	(+3.8%)	(+5.6%)	(+5.3%)

Okapi06c	0.2596	0.0759	0.0601	0.4388
RelNov(Wiki)	0.2705	0.0801	0.0630	0.4616
	(+4.2%)	(+5.5%)	(+4.8%)	(+5.2%)

For TREC 2006 Genomics track topics, re-ranking performances on three baseline runs in terms of Aspect MAP, Passage MAP, Passage2 MAP and Document MAP are shown in this table.

We also note that, in terms of Aspect MAP, the improvements on the 2007’s topics are more significant than the improvements on the 2006’s topics. This might be due to that the average number of distinct aspects of each 2007’s topic (72.3 aspects per topic) is much larger than that of each 2006’s topic (27.9 aspects per topic) [1,2]. A topic with more distinct aspects indicates the information need of this topic could be more diverse. In this case, our approach performs better.

### Effect of the use of aspects

**Figure 3:**
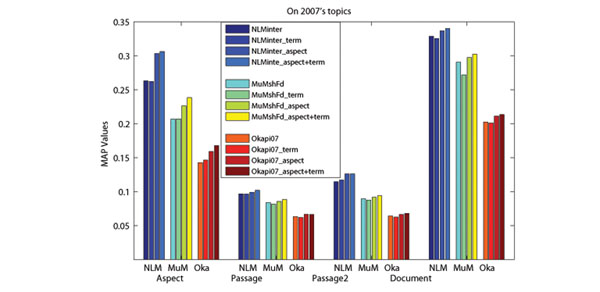
**Effects of the use of aspects on 2007s topics**. The x-axis presents the evaluation measures, where “NLM”, “MuM” and “Oka” in the left figure stand for three baselines corresponding to NLMinter, MuMshFd and Okapi07.

**Figure 4:**
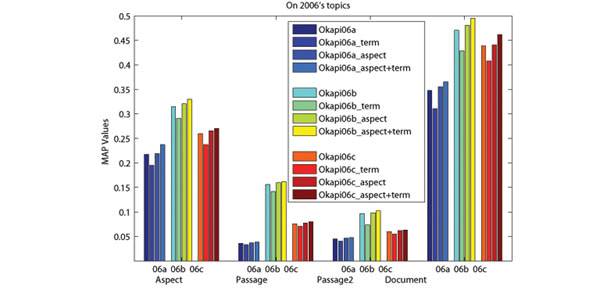
**Effects of the use of aspects on 2006s topics.** The x-axis presents the evaluation measures, where “06a”, “06b” and “06c” in the right figure stand for three baselines corresponding to Okapi06a, Okapi06b and Okapi06c.

In order to capture the conceptual information of retrieved documents, we use Wikipedia concepts to present the aspects covered by retrieved documents. The advantage of using Wikipedia for aspect detection is that Wikipedia not only provides concepts (entities) and lexical variants of a specific term, but also provides abundant contexts. With the help of enriched entity pages, it is possible to identify which concepts and lexical variants are related under a specific context [18]. From Figure 3 and 4, we can see that performance improvements can be achieved when we only use aspects for re-ranking. Nonetheless, the best performances are achieved when both terms and aspects are used. Moreover, re-ranking without aspect (only using terms in retrieved documents) may hurts the retrieval performance. This might be due to the frequent use of (possibly non-standardized) acronyms, the presence of homonyms and synonyms in biomedical literatures. Therefore, using Wikipedia (with enriched contexts provided by entity pages) to detect aspects and presenting them with Wikipedia concepts plays an important role in the re-ranking.

### Compare with aspect detection using UMLS

Our experimental results have demonstrated that aspect detection using Wikipedia is effective in result diversification. However, in biomedical IR, the use of domain-specific thesauri is still the most commonly used method of integrating external knowledge. Therefore, it is worthwhile to compare the re-ranking performance based on aspect detection using Wikipedia and using domain-specific thesauri. We use the largest thesaurus UMLS(http://www.nlm.nih.gov/pubs/factsheets/umls.html) in the biomedical domain as the knowledge resource for aspect detection. In practice, we use MetaMap [35], a program developed at the National Library of Medicine (NLM), to map biomedical text to the thesaurus or, equivalently, to discover thesaurus concepts referred to in text. The UMLS Metathesaurus is used as MetaMap’s biomedical knowledge resource. It includes the NCBI taxonomy, Gene Ontology, the Medical Subject Headings (MeSH), OMIM and the Digital Anatomist Symbolic Knowledge Base. Research showed that MetaMap is an effective tool for discovering thesaurus concepts in text [35].

Re-ranking results based on aspect detection using the UMLS are presented in Table 6 and 7. As we can see, when the UMLS is used for aspect detection, performance improvements can be obtained in terms of Aspect MAP and Passage2 MAP. However, Passage MAP and Document MAP may decrease on some baselines. We also find that, compared with aspect detection using the UMLS, aspect detection based on Wikipedia can achieve more significant and more stable performance improvements. This is because the enriched entity pages in Wikipedia could result in a better mapping between terms in biomedical text and concepts. Moreover, instead of only providing hierarchical relationships (synonyms, hypernyms, hyponyms) among biomedical concepts like the UMLS, plenty of Wikipedia links and anchor texts can also provide more natural relationships among Wikipedia concepts.

**Table 6:** Re-ranking performance on 2007’s topics with aspect detection using UMLS

MAP	Aspect	Passage	Passage2	Document
NLMinter	0.2631	0.0968	0.1148	0.3286
RelNov(UMLS)	0.2962	0.0952	0.1201	0.3264
	(+12.6%)	(-1.6%)	(+4.6%)	(-0.6%)

MuMshFd	0.2068	0.0840	0.0895	0.2906
RelNov(UMLS)	0.2251	0.0862	0.0921	0.2982
	(+8.8%)	(+2.6%)	(+2.9%)	(+2.6%)

Okapi07	0.1428	0.0633	0.0641	0.2025
RelNov(UMLS)	0.1593	0.0648	0.0661	0.2097
	(+11.5%)	(+2.4%)	(+3.1%)	(+3.5%)

For TREC 2007 Genomics track topics, re-ranking performances on three baseline runs in terms of Aspect MAP, Passage MAP, Passage2 MAP and Document MAP are shown in this table.

**Table 7:** Re-ranking performance on 2006’s topics with aspect detection using UMLS

MAP	Aspect	Passage	Passage2	Document
Okapi06a	0.2176	0.0362	0.0450	0.3476
RelNov(UMLS)	0.2235	0.0371	0.0463	0.3551
	(+2.7%)	(+2.5%)	(+2.9%)	(+2.1%)

Okapi06b	0.3147	0.1559	0.0968	0.4705
RelNov(UMLS)	0.3197	0.1545	0.0981	0.4678
	(+1.6%)	(-0.9%)	(+1.3%)	(-0.6%)

Okapi06c	0.2596	0.0759	0.0601	0.4388
RelNov(UMLS)	0.2655	0.0773	0.0621	0.4476
	(+2.3%)	(+1.8%)	(+3.3%)	(+2.0%)

For TREC 2006 Genomics track topics, re-ranking performances on three baseline runs in terms of Aspect MAP, Passage MAP, Passage2 MAP and Document MAP are shown in this table.

### Comparison with the subtopic retrieval method

The subtopic retrieval method proposed by Zhai *et al.* in [5] combined relevance scores from a retrieval baseline with novelty scores from the mixture model using a cost-based method. Their work was based on the maximum marginal relevance (MMR) ranking function [3] and argued for the value of diversity. The subtopic retrieval method was shown to be effective in promoting diversity in the ranked list [5]. In order to further evaluate the proposed approach, we compare it with the subtopic retrieval method.

**Figure 5:**
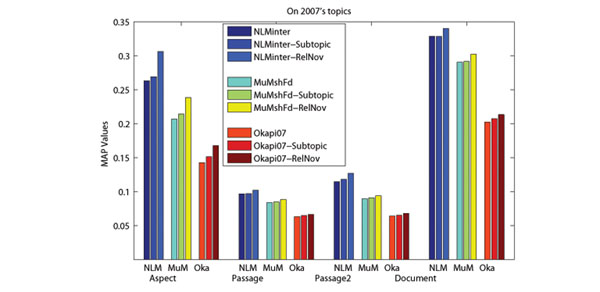
**Comparison with the subtopic retrieval method on 2007’s topics.** The x-axis presents the evaluation measures, where “NLM”, “MuM” and “Oka” in the left figure stand for three baselines corresponding to NLMinter, MuMshFd and Okapi07.

**Figure 6:**
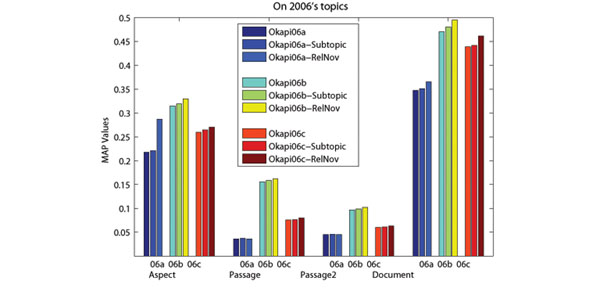
**Comparison with the subtopic retrieval method on 2006’s topics.** The x-axis presents the evaluation measures, where “06a”, “06b” and “06c” in the right figure stand for three baselines corresponding to Okapi06a, Okapi06b and Okapi06c.

The comparison results shown in Figure 5 and 6 illustrate that our approach outperforms the subtopic retrieval method on three levels retrieval. The advantage of our approach is more significant in terms of Aspect MAP. This indicates that our approach is more effective in promoting ranking diversity for biomedical IR. In our approach, aspects covered by retrieved documents are presented by corresponding Wikipedia concepts. On the other hand, the MMR method employs text similarity as the novelty measure, which uses terms in the retrieved documents to compute document novelty. Because of the frequent use of (possibly non-standardized) acronyms, the presence of homonyms and synonyms in biomedical literatures, using Wikipedia (with enriched contexts provided by entity pages) to detect aspects and presenting them with Wikipedia concepts could result in better biomedical IR performance.

## Conclusions

In this paper, we present a relevance-novelty combined model based on an undirected graphical model for promoting ranking diversity in genomics search. The proposed combination model, namely RelNov, models aspects, terms, topic relevance and document novelty as potential functions. Specifically, we propose a two-stage model for modeling aspect-term topic relevance and use the mixture model to measure aspect-term novelty. Experimental results demonstrate that the proposed approach is effective in promoting ranking diversity as well as in improving relevance of ranked lists for genomics search. The use of aspect also plays an important role in the model. Moreover, the proposed model is flexible enough, which can integrate various different relevance and novelty measures easily.

## Competing Interests

The authors declare that they have no competing interests.

## Authors' contributions

XY proposed the relevance-novelty combination framework, performed the experiments and drafted the manuscript. JXH contributed in the study design and experiments. ZL, JXH and XH supervised the study and revised the manuscript.
